# Maternal Omega-3 PUFA Supplementation and Mitochondrial Function in a Newborn Piglet Model: A Preliminary Investigation

**DOI:** 10.3390/ijms27072995

**Published:** 2026-03-25

**Authors:** Paweł Kowalczyk, Monika Sobol, Ewa Święch, Anna Tuśnio, Marcin Barszcz, Jarosław Woliński, Joanna Makulska, Andrzej Węglarz, Grzegorz Skiba

**Affiliations:** 1 Department of Animal Nutrition, The Kielanowski Institute of Animal Physiology and Nutrition, Polish Academy of Sciences, Instytucka 3, 05-110 Jabłonna, Poland; m.sobol@ifzz.pl (M.S.); e.swiech@ifzz.pl (E.Ś.); a.tusnio@ifzz.pl (A.T.); m.barszcz@ifzz.pl (M.B.); 2 Large Animal Models Laboratory, Department of Animal Physiology, The Kielanowski Institute of Animal Physiology and Nutrition, Polish Academy of Sciences, Instytucka 3, 05-110 Jabłonna, Poland; j.wolinski@ifzz.pl; 3 Department of Genetics, Breeding and Animal Ethology, Hugo Kołłątaj University of Agriculture in Krakow, Adama Mickiewicza 21, 31-120 Kraków, Poland; joanna.makulska@urk.edu.pl (J.M.); andrzej.weglarz@urk.edu.pl (A.W.)

**Keywords:** maternal nutrition, mitochondria, omega-3 fatty acids, piglet model, algal oil, fish oil, oxidative DNA damage, oxidative phosphorylation

## Abstract

Maternal nutrition during pregnancy plays a crucial role in fetal development and metabolic programming. Long-chain omega-3 polyunsaturated fatty acids (LC-PUFA n-3), particularly eicosapentaenoic acid (EPA) and docosahexaenoic acid (DHA), are known to influence mitochondrial function and cellular energy metabolism. The present preliminary study aimed to evaluate the effects of maternal omega-3 supplementation on mitochondrial bioenergetics in neonatal piglets. Pregnant sows were supplemented with either fish oil or algal oil rich in LC-PUFA n-3 (long-chain omega-3 polyunsaturated fatty acids) throughout gestation. Liver samples were collected from newborn piglets immediately after birth, and mitochondrial respiratory parameters, oxygen consumption rates, and selected oxidative stress markers were analyzed. The results indicated that maternal omega-3 supplementation was associated with improved mitochondrial respiratory parameters and enhanced oxidative phosphorylation efficiency in neonatal liver tissue. Both fish oil and algal oil supplementation showed similar trends in improving mitochondrial bioenergetic function. Although the study was exploratory and conducted on a limited number of animals, the findings suggest that maternal intake of LC-PUFA n-3 may influence mitochondrial metabolism in offspring. Further studies with larger experimental groups are required to confirm these observations and to better understand the mechanisms underlying these effects.

## 1. Introduction

Mitochondria play a key role in cellular bioenergetics and metabolic homeostasis, serving as the primary sites of energy production in the form of ATP through oxidative phosphorylation via the respiratory chain [1,2,3,4]. They are also involved in lipid metabolism regulation, the generation and neutralization of reactive oxygen species (ROS), and the initiation of cell death [2]. Therefore, the assessment of mitochondrial function represents an essential element of studies on metabolic health, energy efficiency, and adaptation to environmental and nutritional changes [3,4]. Mitochondrial dysfunction can affect growth, stress resistance, and metabolic development in neonates; thus, evaluating mitochondrial parameters (e.g., oxygen consumption rate, spare respiratory capacity) is of crucial importance in nutritional and physiological studies of livestock [4]. Livestock nutrition, including that of sows, has a profound impact on maternal metabolic systems as well as on the development and health of their offspring [5].

Omega-3 long-chain polyunsaturated fatty acids (LC-PUFA n-3), particularly eicosapentaenoic acid (EPA) and docosahexaenoic acid (DHA), play an important role in regulating inflammatory responses and oxidative stress. These fatty acids can modulate cellular signaling pathways, including NF-κB and PPAR-mediated mechanisms, thereby influencing mitochondrial membrane composition, respiratory efficiency, and redox homeostasis. Consequently, maternal dietary intake of LC-PUFA n-3 during pregnancy may affect fetal metabolic programming and mitochondrial development in the offspring [6,7,8,9,10,11,12,13,14,15,16,17].

In recent years, particular attention has been paid to the role of marine-derived fatty acids, especially long-chain polyunsaturated omega-3 fatty acids (LC-PUFA n-3) found in fish and algal oils [6,7]. These compounds exhibit anti-inflammatory properties, modulate the expression of genes related to energy metabolism, and may influence mitochondrial biogenesis and function in skeletal muscle, cardiac tissues, and hepatocytes [7,8,9,10]. The inclusion of these lipid sources in sow diets may therefore affect not only maternal metabolism but also the metabolic development and mitochondrial function of piglets during the prenatal and postnatal periods [11,12].

In the context of swine production, modifying the dietary fatty acid profile by increasing the proportion of LC-PUFA n-3, such as EPA and DHA, is of great interest due to its potential health and metabolic benefits [10,11,12,13]. Recently, special attention has been focused on maternal supplementation with these bioactive lipids [14,15,16,17]. Such dietary interventions alter the fatty acid composition of milk and offspring tissues, potentially influencing brain development, immune function, and energy metabolism in neonates [18]. Mechanistically, LC-PUFA n-3 are incorporated into mitochondrial membrane phospholipids, which modulates membrane fluidity, affects the function of electron transport proteins, and regulates the expression of genes involved in oxidative metabolism and mitochondrial biogenesis (e.g., PPARα, PGC-1α) [19,20,21,22].

Research in pigs has demonstrated changes in the expression of genes associated with lipid metabolism and mitochondrial function in offspring [23,24,25,26,27], as well as modulation of DNA damage and base excision repair (BER) enzyme activity in piglets born from sows supplemented during pregnancy with algal and fish oils rich in LC-PUFA n-3 [23,24]. This suggests a potential impact on mitochondrial function in organs such as muscles and the liver [23,24,25,26,27,28]. At the same time, omega-3 fatty acid supplementation may influence redox balance, although results vary depending on the dose, source (fish vs. algal oil), and dietary antioxidant content [21,29]. A growing body of evidence indicates that gestational supplementation with LC-PUFA n-3 reduces oxidative stress markers and DNA damage in neonates, potentially improving metabolic parameters during the early postnatal period [23,24,29].

Our most recent studies have compared the effects of fish oil and algal oil (microalgal) supplementation, including innovative formulations such as encapsulation, regarding bioavailability and biological outcomes in neonatal piglets [23,24]. However, most available studies have focused primarily on phenotypic parameters, fatty acid profiles, and inflammatory markers—lacking a comprehensive, systematic evaluation of mitochondrial function in the tissues of newborn piglets [28].

Therefore, there is a methodological and conceptual gap in linking precise measurements of offspring oxygen consumption with nutritional studies of sows receiving n-3 LC-PUFA supplementation during pregnancy. Such studies not only demonstrate alterations in offspring lipid profiles but also allow for an assessment of how these changes translate into respiratory chain performance, mitochondrial efficiency, and susceptibility to oxidative stress in metabolically key organs, such as the liver [1,3,4,12,29]. Clarifying these mechanisms has both practical and theoretical implications, potentially supporting the optimization of maternal nutrition strategies and enhancing understanding of metabolic “programming” of offspring through maternal diet [1,2,3,4,5,9,10,11,12,13,14,15,16,17,18,19,20,21,22,23,24].

Maternal nutrition during pregnancy is a critical determinant of fetal development and long-term metabolic health [1,2,3,4,5,9,10,11,12,13,14,15,16,17,18,19,20,21,22,23,24]. Increasing evidence suggests that dietary components consumed during gestation can influence fetal metabolic programming and may affect mitochondrial development and function in the offspring. Long-chain omega-3 polyunsaturated fatty acids (LC-PUFA n-3), particularly eicosapentaenoic acid (EPA) and docosahexaenoic acid (DHA), play essential roles in cellular metabolism, membrane structure, and mitochondrial bioenergetics [10,11,12,13,14,15,16,17]. These fatty acids are incorporated into mitochondrial membranes, where they may influence membrane fluidity, electron transport chain activity, and oxidative phosphorylation efficiency. In addition to their structural role in cellular membranes, LC-PUFA n-3 exhibit well-documented anti-inflammatory and antioxidative properties [10,11,12,13,14,15,16,17]. Omega-3 fatty acids can modulate key inflammatory signaling pathways, including NF-κB and PPAR-mediated mechanisms, thereby reducing the production of pro-inflammatory mediators and reactive oxygen species. Through these mechanisms, omega-3 fatty acids may contribute to improved mitochondrial efficiency and redox homeostasis. During pregnancy, maternal dietary intake of LC-PUFA n-3 is particularly important, as these fatty acids are actively transferred to the developing fetus and play a crucial role in organ development, including the brain and liver. However, relatively little is known about the influence of maternal omega-3 supplementation on mitochondrial function in the offspring. The pig is considered a valuable animal model for human physiology due to similarities in metabolism, digestive processes, and organ development. Therefore, the present study aimed to investigate the effects of maternal supplementation with omega-3 fatty acids derived from fish oil or algal oil on mitochondrial respiratory function in the liver of newborn piglets [2,23,24,25,26,27,28,29].

Thus, the aim of the present study was to evaluate liver mitochondrial function in neonatal piglets in response to gestational supplementation of sows with different LC-PUFA n-3 sources.

## 2. Results

### 2.1. Oxygen Consumption

**Figure 1:**
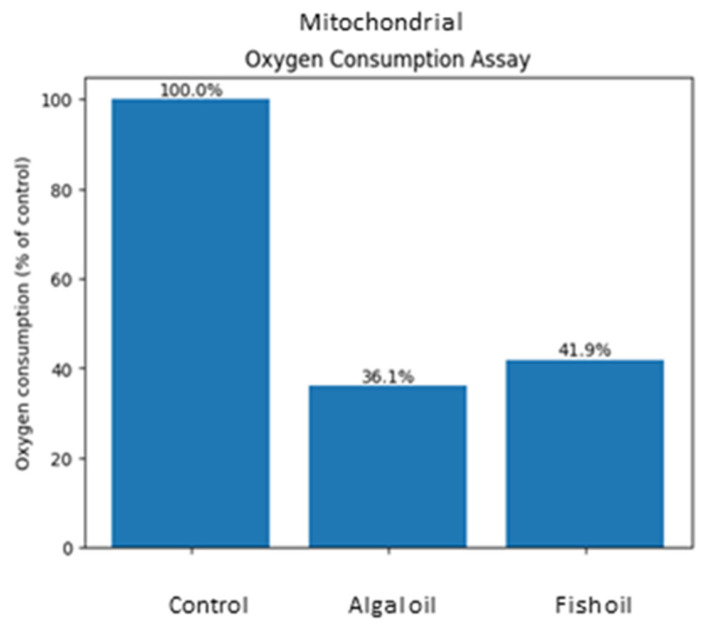
Mitochondrial oxygen consumption rate (OCR), (pmol O_2_/min/mg protein) in the liver tissue of newborn piglets (first day of life) born from the control sow or sows supplemented during pregnancy with fish or algal oil. Values for experimental groups are expressed as % of the control piglets. Bars were created as a mean value for 6 samples in each group.

The results of oxygen consumption assay are shown in Figure 1. Both fish and algal oil supplementation during pregnancy sows diet enhanced mitochondrial respiratory efficiency in the liver of their offspring. This was evidenced by a reduction in oxygen consumption in these groups compared to the control (Figure 1).

### 2.2. Mitochondrial Respiration Assessed by Oroboros Oxygraph 2k

Mean values of mitochondrial respiration parameters differed between litters. Piglets born from sows supplemented with fish oil or algal oil exhibited higher mean ROUTINE respiration compared to the control litter. Proton leak showed comparable values across groups, with slightly lower means observed in supplemented litters. OXPHOS capacity demonstrated higher mean values in piglets from both supplemented groups relative to control. A similar pattern was observed for ETS capacity, where the fish oil and algal oil litters displayed greater mean electron transport system activity. In contrast, LEAK respiration did not differ significantly between groups. Respiratory control ratios indicated a tendency toward improved coupling efficiency in piglets born to supplemented sows. The ROUTINE/LEAK ratio was numerically higher in both supplemented groups, while the LEAK/OXPHOS ratio showed slightly lower mean values compared to control. The OXPHOS/ETS ratio remained comparable across groups (Table 1).

### 2.3. Fluorespirometry

Mitochondrial oxygen consumption rate analysis in the liver of newborn piglets, measured using fluorespirometry, showed significant differences between the experimental and control groups (Table 2 and Table 3). Both algal and fish oil groups exhibited similar but significantly higher (*p* < 0.05) basal respiration compared to the control group. Similar trend was observed for ATP-linked respiration. In turn, maximal respiration and spare respiratory capacity were the highest (*p* < 0.01) in piglets from sows supplemented with fish oil, followed by algal oil groups, and the lowest in the control group. However, proton leak values did not differ significantly between groups.

### 2.4. Correlations Between Parameters of Mitochondrial Functions and Markers of Oxidative DNA Damage

Pearson’s correlation analysis (Table 4) revealed moderate positive relationship between 8-oxo-deoxyguanosine (8-oxo-G) and both basal and maximal respiration (r = 0.52, *p* = 0.032; r = 0.58, *p* = 0.021, respectively). In contrast, 8-oxo-G was negatively correlated (*p* < 0.05) with spare respiratory capacity (r = −0.41). Regarding the 1,N^6^-etheno-2′-deoxyadenosine (εA) and 3,N^4^-etheno-2′-deoxycytidine (εC) markers, only the proton leak parameter was significantly correlated (*p* < 0.05) with these indicators (r = 0.47 and r = 0.43, respectively).

## 3. Discussion

The present study investigated the potential effects of maternal omega-3 fatty acid supplementation on mitochondrial bioenergetics in neonatal piglets. Our results suggest that maternal dietary intake of LC-PUFA n-3 may influence mitochondrial respiratory parameters and oxidative balance in the liver of newborn offspring.

One possible explanation for these observations is the incorporation of omega-3 fatty acids into mitochondrial membranes. Omega-3 fatty acids are long-chain polyunsaturated fatty acids (LC-PUFA n-3), mainly represented by eicosapentaenoic acid (EPA) and docosahexaenoic acid (DHA), which are known to modify membrane lipid composition. Their incorporation into mitochondrial membranes may alter membrane fluidity and influence the activity of proteins involved in the electron transport chain, thereby improving mitochondrial respiration and oxidative phosphorylation efficiency [30,31,32,33,34,35,36,37,38,39,40,41,42,43,44,45,46]. In addition to these structural effects, omega-3 fatty acids exhibit well-documented anti-inflammatory and antioxidative properties. EPA and DHA can modulate inflammatory signaling pathways, including NF-κB- and PPAR-mediated mechanisms, leading to reduced production of pro-inflammatory cytokines and reactive oxygen species as well as improved cellular redox homeostasis [30,31,32,33,34,35,36,37,38,39,40,41,42,43,44,45]. Through these mechanisms, omega-3 fatty acids may indirectly support mitochondrial function, enhance mitochondrial bioenergetics, and improve respiratory capacity by stabilizing mitochondrial membranes and increasing the efficiency of the electron transport chain [29,38,39,40,41,42,43,44,45,47,48,49,50,51,52,53,54,55]. Consequently, the improved mitochondrial respiratory parameters observed in the present study may partly reflect the combined structural, anti-inflammatory, and antioxidative actions of LC-PUFA n-3 [29,30,31,32,33,34,38,39,40,41,42,43,44,45,46,47,48,49,50,51,52,53,54,55].

Interestingly, similar trends were observed in both the fish oil and algal oil supplementation groups. Although the fatty acid profiles of these oils differ slightly, both provide significant amounts of biologically active LC-PUFA n-3 [55,56,57,58,59,60,61,62,63,64,65]. The comparable mitochondrial responses observed in this study may therefore reflect the availability of DHA and other long-chain omega-3 fatty acids supplied through maternal nutrition.

It should also be noted that the algal oil formulation used in this study contained olive oil as a carrier lipid. Olive oil naturally contains bioactive compounds such as tocopherols and polyphenols, which possess antioxidant properties. Although the present study focused primarily on LC-PUFA n-3, the potential contribution of these additional compounds to oxidative balance cannot be completely excluded and should be considered in future investigations [29,38,39,40,41,42,43,44,45,46,47,48,49,50,51,52,53,54,55].

Long-chain omega-3 fatty acids such as EPA and DHA have been shown to influence mitochondrial bioenergetics and cellular redox balance in various experimental and clinical models [40,41,42]. These fatty acids may modulate mitochondrial membrane properties, oxidative phosphorylation efficiency and antioxidant defense mechanisms [66,67], while supplementation studies have demonstrated improvements in mitochondrial function and reductions in inflammatory responses [42,43,44,45,46,47]. Moreover, maternal omega-3 status during pregnancy may influence fetal metabolic programming and developmental outcomes in the offspring [43,44,45,46,68,69,70,71,72,73,74,75].

The pig is widely regarded as a valuable model for studies of human metabolism due to similarities in digestive physiology, lipid metabolism, and organ development. Therefore, the present findings may contribute to a better understanding of the potential role of maternal omega-3 fatty acid intake in shaping mitochondrial metabolism during early development [46,68,72,73,74,75].

Results of the presented study indicate that mitochondrial oxidative phosphorylation and electron transport system capacities were significantly increased in piglets born from sows receiving either fish or algal oil during pregnancy [16,29,47,48,49,50,51,52,53,54,55]. Elevated OXPHOS and ETS activities reflect enhanced capacity for ATP synthesis and electron flow, both of which are essential for supporting rapid postnatal growth, thermoregulation, and metabolic adaptation to extrauterine life. Furthermore, the improved respiratory control ratio observed in this study confirm enhanced coupling between substrate oxidation and ATP synthesis, suggesting more efficient mitochondrial energy transduction [16,29,47,48,49,50,51,52,53,54,55].

Measuring oxygen consumption rate in isolated mitochondria or living tissue/cell preparations is a well-established, sensitive, and quantitative approach to studying mitochondrial function. Technologies such as Seahorse XF, microplate OCR systems, and high-resolution respirometry-Oroboros allow for the measurement of basal, ATP-linked, resting, and maximal respiration (following uncoupler application), as well as spare respiratory capacity. These are critical parameters for assessing bioenergetic efficiency and susceptibility to dysfunction. Such methods have been optimized for both isolated mitochondria and tissue fragments and are widely used in studies investigating dietary interventions that modulate bioenergetics [14,16,29,47,48,49,50,51,52,53,54,55].

The results of the present study demonstrate that spare respiratory capacity (SRC) was markedly elevated in piglets born from supplemented sows. High SRC reflects the ability of cells to increase ATP production in response to additional energy demands, which is particularly crucial during the neonatal period for maintaining homeostasis and supporting anabolic processes [68,76,77,78,79,80,81,82]. Similar observations have been reported in studies with other species, where DHA and EPA supplementation was associated with improved oxidative phosphorylation and greater bioenergetic reserve [10,83]. Importantly, these changes were observed in liver following both fish and algal oil supplementation, suggesting systemic mitochondrial adaptations [30,31,32,33,34].

Interestingly, results of the present study indicate that supplementation with either fish or algal oil resulted in comparable increases in mitochondrial respiratory capacity. This suggests that algal oil-derived DHA is as equally effective as marine fish oil in modulating fetal mitochondrial development and functions. Algal oil provides a direct, contaminant-free source of DHA with high bioavailability, which may explain its comparable efficacy. Given the environmental and sustainability concerns associated with marine fish oil extraction, algal oil represents a promising alternative for improving sow milk quality and piglet health [35,36,37,66,67,84,85,86].

It should also be noted that the vegan formulation of the supplement contained algal oil combined with olive oil as a carrier lipid. Olive oil naturally contains bioactive compounds such as tocopherols (vitamin E) and polyphenols, which possess antioxidant properties [38,39,40,41,42,43,44,45,46]. Although the present study focused primarily on the effects of LC-PUFA n-3 on mitochondrial bioenergetics, the presence of additional antioxidant compounds in the algal oil preparation cannot be completely excluded as a contributing factor influencing oxidative balance. Future studies should therefore consider a more detailed compositional analysis of dietary supplements to better distinguish the individual contributions of these components [38,39,40,41,42,43,44,45,46,56,57,58,59,60,61,62,63,64,65,68,69,70,71,72,73,74,75].

Present study demonstrates that enhanced mitochondrial efficiency in offspring was confirmed by reduced oxygen consumption per unit of ATP produced, indicating improved coupling efficiency and lower proton leak. The lack of significant differences in LEAK respiration across dietary groups suggests that the observed improvements in respiratory efficiency were not associated with increased membrane proton permeability or mitochondrial uncoupling. These effects were probably mediated by structural and functional remodeling of mitochondrial membranes induced by the incorporation of long-chain PUFA n-3. Both DHA and EPA are known to optimize respiratory supercomplex organization and reduce ROS formation, thereby improving electron transfer efficiency [56,57,58,59,60,61,62,63,64,65]. Generally, the effects of long-chain PUFA n-3 on mitochondria are tissue-specific but systemic. Their incorporation into membranes enhances fluidity, stabilizes respiratory supercomplexes, and promotes efficient electron transport. These modifications support higher ATP yield per oxygen consumed and reduce proton leak, which is critical for neonates transitioning to extrauterine life [87,88,89,90,91]. Additionally, improved mitochondrial performance may influence endocrine and metabolic signaling pathways, including insulin sensitivity, lipid metabolism, and thermoregulation, further contributing to offspring health and production efficiency [92,93,94,95,96].

In the present study, correlations between mitochondrial respiratory parameters and DNA damage were also established. Pearson’s correlation coefficients highlight the physiological relevance of improved mitochondrial function. Positive associations between basal and maximal respiration and 8-oxo-G levels suggest that increased oxidative metabolism may be accompanied by elevated oxidative DNA damage. However, the negative correlation observed between spare respiratory capacity and 8-oxo-G indicates that higher mitochondrial reserve may confer protection against oxidative stress, allowing mitochondria to meet energy demands without excessive ROS generation, as previously discussed [15,16,33,34,35,36,37,56,57,58,59,60,61,62,63,64,65,66,67,84,85,86,87,88]. Furthermore, correlations between proton leak and etheno-DNA adducts (εA and εC) emphasize the importance of mitochondrial membrane composition in maintaining redox homeostasis [23,24].

Literature evidence suggests that the beneficial effects of maternal pregnant supplementation with LC-PUFA n-3 on offspring mitochondrial function likely result from enhanced placental transfer of DHA and EPA and their subsequent incorporation into fetal hepatic mitochondrial membranes. These fatty acids are integral components of cardiolipin, a mitochondrial-specific phospholipid essential for optimal respiratory chain function and supercomplex stabilization [9,60,61,62,63,64,65,87,88,89,90]. DHA-enriched cardiolipin improves respiratory efficiency, reduces electron leakage, and promotes mitochondrial biogenesis [10,18]. Maternal DHA supplementation has also been shown to modulate fetal gene expression related to oxidative metabolism, inflammation, and antioxidant defense, suggesting epigenetic programming mechanisms [4,19,91].

Although the fatty acid profiles of fish oil and algal oil differ slightly in their EPA and DHA proportions, the daily supplementation dose was calculated to provide a comparable supply of DHA to the animals. This approach was chosen because DHA is considered one of the most biologically relevant LC-PUFA n-3 involved in fetal development and mitochondrial membrane composition [4,9,19,60,61,62,63,64,65,87,88,89,90,91].

From a production and physiological perspective, enhanced mitochondrial bioenergetics in offspring may translate into improved growth performance, immune competence, and postnatal robustness—factors that are crucial for early postnatal growth. Previous studies have demonstrated that maternal supplementation with fish oil or algal extracts improves piglet immunity, reduces post-weaning diarrhea, enhances gut integrity, and improves growth performance [6,7,20,21,22,23,24,66,67,85,86]. Improved mitochondrial function likely represents a central mechanistic pathway underlying these benefits, as mitochondrial activity (functions) is fundamental to immune cell function, intestinal barrier maintenance, and metabolic homeostasis [12,28].

Importantly, enhanced mitochondrial bioenergetics may also be a basement for long-term programming of energy metabolism in offspring. Supplementation of sows with LC-PUFA n-3 during pregnancy can improve ATP production efficiency and reserve capacity in newborn offspring. Furthermore, the use of such additives in sow nutrition can reduce neonatal vulnerability to metabolic stressors, such as nutrient fluctuations, oxidative challenges, or immune activation during weaning and early growth [1,2,3,4,5,6,7,8,9,10,11,12,13,14]. Such metabolic programming may contribute to a better feed conversion ratio, faster growth rate, and overall increased immunity, thereby optimizing production outcomes throughout the postnatal growth period [28,29,35,47,48,49]. Moreover, mitochondrial efficiency influences not only energy metabolism but also the regulation of redox homeostasis and inflammatory responses. Offspring with higher spare respiratory capacity can buffer ROS generation during periods of high metabolic demand, protecting tissues from oxidative damage and supporting immune competence [15,16,19,28]. These effects may partially explain the observed reductions in oxidative DNA damage markers and improvements in liver antioxidant defenses in supplemented groups [15,16,17,18,19,20,21,22,23,24,34,35,36,37,56].

From a translational perspective, the comparable efficacy of algal and fish oils underscores the potential of sustainable, contaminant-free sources of LC-PUFA n-3 in animal nutrition. Algal oil supplementation may serve as a practical alternative in commercial sow diets, maintaining bioenergetic programming benefits while reducing ecological impacts associated with marine fish oil extraction [21,22,23,24,25,26,27,28,29,47,48,49,50,51,52,53,54,55,68,76,77,78]. Moreover, early-life mitochondrial programming may have multigenerational implications, as improved neonatal metabolism and growth can influence reproductive performance and overall herd productivity [35,36,37,46,66,67,68,75,84].

From a translational perspective, the supplementation dose applied in the present experiment was estimated based on recommended DHA intake levels during human pregnancy. However, direct extrapolation of dosing between species must be interpreted cautiously due to differences in metabolic rate, body mass, and reproductive physiology. Nevertheless, the present findings support the growing body of evidence suggesting that adequate maternal intake of LC-PUFA n-3 may positively influence mitochondrial development and metabolic programming in offspring [1,2,3,4,5,6,7,8,9,10,11,12,13,14,15,16].

Despite these limitations, the present study provides novel preliminary evidence that maternal supplementation with long-chain omega-3 polyunsaturated fatty acids during pregnancy may influence mitochondrial bioenergetic parameters and oxidative balance in the liver tissue of newborn piglets [12,28]. The results suggest that maternal dietary intake of LC-PUFA n-3 could contribute to early metabolic programming by modulating mitochondrial respiration and cellular redox homeostasis in the offspring. Given the physiological similarities between pigs and humans in terms of lipid metabolism and organ development, the piglet model represents a valuable experimental system for investigating the effects of maternal nutrition on neonatal metabolic function [35,49]. Therefore, the findings of this study may contribute to a better understanding of the potential role of maternal omega-3 fatty acid intake in shaping mitochondrial metabolism during early development. Nevertheless, further studies involving larger experimental groups and comprehensive maternal metabolic characterization are required to confirm these observations and to elucidate the underlying molecular mechanisms.

## 4. Materials and Methods

### 4.1. Animals, Housing and Feeding

The study involved three primiparous Polish Landrace sows (gilts) purchased from the Animal Breeding Center in Chodeczek, Poland.

The study was designed as a preliminary exploratory experiment aimed at evaluating potential effects of maternal LC-PUFA n-3 supplementation on mitochondrial function in neonatal piglets. Due to the exploratory nature of the study, three primiparous Polish Landrace sows (gilts) were used, each representing a different dietary treatment groups (control, fish oil, or algal oil supplementation).

Following pregnancy confirmation, the animals were transported to the Large Animal Models Laboratory of the Institute and allowed to acclimatize to the housing conditions. After the adaptation period, each sow was assigned to one dietary treatment: control diet (no supplementation), diet supplemented with fish oil, or diet supplemented with algal oil rich in LC-PUFA n-3.

The supplementation started immediately after pregnancy confirmation and continued throughout the gestation period until parturition.

Animals were fed a standard diet adjusted to their gestational stage (early or late pregnancy) according to NRC recommendations. Feeding was performed using controlled daily rations, which were fully consumed by the animals, ensuring comparable feed intake between the experimental groups. Until day 90 of pregnancy, sows were housed individually, after which they were transferred to farrowing pens under controlled environmental conditions.

After an adaptation period, gilts were fed a standard diet adjusted to their gestational stage (early or late pregnancy) in accordance with NRC recommendations [12]. All diets were balanced for energy, protein, amino acids, and vitamins. As the ingredient composition of the commercial diets was proprietary, only their nutritional values are presented (Table 5). After pregnancy confirmation, each sow was assigned to a different dietary treatment involving supplementation with fish oil (sow no. 2) or algal oil (sow no. 3) rich in LC-PUFA n-3, or to an unsupplemented control diet (sow no. 1). Both fish and algal oils were added individually to the daily rations immediately before feeding. The daily dose of natural oils was calculated to provide each sow with 3100 mg of DHA per day, accounting for the maternal requirement (600 mg) and fetal needs (250 mg per fetus, assuming 10 fetuses). Due to the lack of specific dietary recommendations for DHA in pregnant sows, the daily dose was estimated based on WHO recommendations for pregnant women. Oils were purchased from NORSAN Poland LLC, a certified producer of Arctic cod and algal oils. The fatty acid composition of the oils is given in Table 6. Until day 90 of pregnancy, the sows were housed in individual pens (2.75 m^2^), and thereafter in farrowing pens (5.5 m^2^), equipped with a rubber mats, a feeder, and nipple drinkers. Central heating and air conditioning systems (Fancom, model ISM0.12; Fancom BV, Panningen, The Netherlands) in the piggery made it possible to keep the animals under thermo-neutral conditions.

Piglets were euthanized within the first 24 h of life using a sodium pentobarbital (Exagon, 400 mg/mL) injected into the marginal ear vein at a dose of 0.1 mL/kg body weight diluted 1:1 with sterile 0.9% NaCl. Liver samples were collected immediately post-mortem. Although the anticipated litter size was estimated at 10 fetuses per sow, the actual litter size was smaller.

Although the expected litter size was approximately 10 fetuses per sow, the actual number of piglets born was lower. Therefore, liver samples were collected from six piglets per litter, resulting in six biological samples per maternal dietary treatment groups. Piglets were selected randomly within each litter to minimize potential litter-related bias.

**Figure 2:**
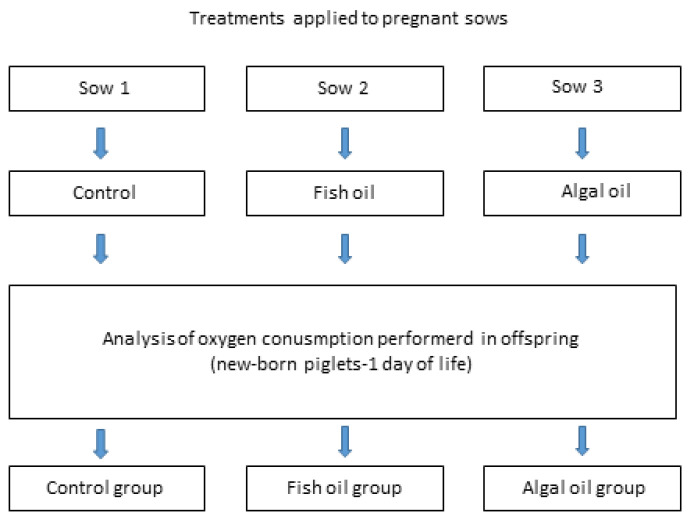
Experimental design. Analysis of mitochondrial oxygen consumption rate (OCR) parameters measured in liver tissue of newborn piglets born to control sows or sows supplemented with fish oil or algal oil during pregnancy.

The experimental design is shown on (Figure 2).

### 4.2. Preparation of Liver Samples for Analyses

Fresh liver samples were collected immediately after euthanasia and placed in ice-cold isolation buffer. The tissue fragments were rinsed in ice-cold phosphate-buffered saline (PBS) to remove residual blood, blotted dry and weighed. Subsequently samples were mechanically homogenized (1:10, *w*/*v*) in ice-cold isolation buffer (100 mmol/L KCl, 50 mmol/L MOPS, 1 mmol/L EGTA, 5 mmol/L MgCl_2_, 1 mmol/L ATP, 0.2% BSA; pH 7.4) using a glass–Teflon homogenizer on ice to preserve mitochondrial integrity. The homogenate was centrifuged at 800× *g* for 10 min at 4 °C to remove cellular debris and nuclei. The resulting supernatant, containing mitochondria-enriched fractions, was used immediately for oxygen consumption measurements. Protein concentration was determined using the Bradford method and used for normalization of mitochondrial respiration rates.

### 4.3. Oxygen Consumption Assay

Oxygen consumption rate (OCR) was measured using a filter-based multi-mode microplate reader (FLUOstar OPTIMA, BMG Labtech, Offenburg, Germany) and MitoXpress-Xtra HS kits (Luxcel Biosciences, Cork, Ireland), according to the manufacturer’s protocol. Fluorescence intensity was recorded at 10 min intervals over a 90 min period at 37 °C.

### 4.4. Substrate–Uncoupler–Inhibitor Titration (SUIT)

Liver samples were prepared as described above. Measurements were performed following the Substrate–Uncoupler–Inhibitor Titration (SUIT) protocol [16,29,55]. Mitochondrial respiration, (Routine, LEAK, OXPHOS, and ETS capacities) was assessed using an Oroboros Oxygraph-2k high-resolution respirometer (Oroboros Instruments, Innsbruck, Austria) by adding substrates and inhibitors: oligomycin (1 µg/mL), FCCP (0.5 µmol/L) and antimycin A (2.5 µmol/L) [16,29]. All respirometric data were normalized to the total protein content determined according to Bradford method [16,29].

### 4.5. High-Resolution Fluorespirometry

Mitochondrial function was assessed using high-resolution respirometry (Oroboros O2k-FluoRespirometer, Innsbruck, Austria), which provides precise, real-time measurement of oxygen consumption rates [16,29,55]. By utilizing liver samples prepared as described above, the respiratory chain was examined under conditions closely reflecting physiological status. This system allows for a comprehensive evaluation of mitochondrial energy efficiency, especially basal respiration, ATP-linked respiration, maximal respiratory capacity, spare respiratory capacity, and proton leak, while simultaneously monitoring oxidative stress parameters when required [5,9,12,55].

### 4.6. Statistical Evaluation

Statistical analysis was performed using GraphPad Prism software version 10.0.0 (GraphPad Software Inc., San Diego, CA, USA). Data are presented as mean ± standard deviation (SD). Differences between experimental groups were evaluated using one-way analysis of variance (ANOVA) followed by Tukey’s post hoc test. In the present exploratory study, six piglets were sampled from each litter representing the maternal dietary treatment.

Because piglets originated from the same maternal environment, the results should be interpreted with caution, and the litter effect cannot be fully excluded. Therefore, the study should be considered preliminary, and the results are intended to identify potential trends in mitochondrial responses to maternal LC-PUFA n-3 supplementation. A *p*-value < 0.05 was considered statistically significant.

## 5. Conclusions

The present preliminary study suggests that maternal supplementation with long-chain omega-3 polyunsaturated fatty acids during pregnancy, derived from either fish or algal oil, may influence mitochondrial bioenergetic processes and oxidative balance in the liver tissue of newborn piglets. Both supplementation strategies were associated with favorable changes in selected indicators of mitochondrial respiration, and the results suggest that algal oil may exert effects comparable to those observed for fish oil.

However, due to the exploratory design of the study and the limited number of maternal animals, these findings should be interpreted with caution. Although the results point to potential metabolic benefits of maternal LC-PUFA n-3 intake during gestation, further research involving larger experimental groups and comprehensive maternal metabolic characterization is required to confirm these observations.

Overall, the present findings add to the growing body of evidence indicating that adequate maternal omega-3 fatty acid intake may play a role in shaping metabolic processes during early development.

## 6. Limitations of the Study

The present study has several limitations that should be considered when interpreting the results. First, the sample size and number of maternal animals and replicates were limited, which may reduce the statistical power to detect subtle differences in certain mitochondrial parameters in offspring. Second, the analysis focused primarily on specific tissues and time points; therefore, potential systemic or developmental effects on other organs may not have been fully captured. Third, although measurements of oxygen consumption rates provide valuable insights into mitochondrial respiration (piglets originated from the same litters within each dietary groups, which may introduce a potential litter-related effect), they do not fully reflect other functional aspects such as membrane potential, ROS production, or ATP synthesis efficiency. Furthermore, the study did not include a direct assessment of mitochondrial morphology or dynamics using imaging techniques, which could have complemented the functional data.

Another limitation is that maternal plasma fatty acid profiles and metabolic parameters were not measured in the present experiment. Such data would provide valuable confirmation of the biochemical effects of dietary supplementation and allow a more direct link between maternal lipid status and offspring mitochondrial function. Future studies should therefore include a larger number of animals and a more comprehensive characterization of maternal metabolic status.

## Figures and Tables

**Table 1:** Parameters of mitochondrial respiration in a newborn piglet model (first day of life) born to sows receiving gestational supplementation with fish or algal oil.

Item	Control	Fish Oil	Algal Oil	SE Pooled	*p*-Value
ROUTINE, pmol O_2_·s^−1^·mg^−1^	38.3 ^a^	44.9 ^b^	46.2 ^b^	2.97	0.0150
LEAK, pmol O_2_·s^−1^·mg^−1^	14.9	13.6	13.3	0.83	0.2405
OXPHOS, pmol O_2_·s^−1^·mg^−1^	77.6 ^a^	89.8 ^b^	91.7 ^b^	4.67	0.0220
ETS pmol O_2_·s^−1^·mg^−1^	112.4 ^a^	129.1 ^b^	131.5 ^b^	5.63	0.0180
Values of respiratory control ratio					
Routine/leak	2.57 ^A^	3.30 ^B^	3.47 ^B^	0.27	0.0092
Leak/OXOPHOS	0.19 ^B^	0.15 ^A^	0.15 ^A^	0.02	0.0089
OXOPHOS/ETS	0.69	0.70	0.70	0.04	0.8101

SE pooled—standard error of the mean; ^a,b^—values within a row with different superscripts indicate statistically significant differences (*p* < 0.05), ^A,B^—values within a row with different superscripts indicate statistically significant differences (*p* < 0.01); ROUTINE—physiological oxygen consumption in intact cells; LEAK—proton leak; OXPHOS—capacity of the oxidative phosphorylation system; ETS—maximum capacity of the electron transport system. The values given in the table are the means calculated from 6 samples in each group.

**Table 2:** Parameters of mitochondrial function (pmol O_2_/min/µg protein), measured using the fluororespirometry method, in the liver of newborn piglets (first day of life) from the control sow and sows supplemented during pregnancy with fish or algal oil.

Item	Control	Fish Oil	Algal Oil	SE Pooled	*p*-Value
Basal respiration	95 ^a^	112 ^b^	105 ^b^	11.53	0.0265
ATP-linked respiration	62 ^a^	71 ^b^	68 ^b^	7.15	0.0311
Proton leak	12	13	12	1.62	0.4839
Maximal respiration	150 ^a^	180 ^c^	168 ^b^	15.08	0.0190
Spare respiratory capacity	55 ^a^	75 ^c^	63 ^b^	7.68	0.0153

SE pooled—standard error of the mean; ^a,b,c^—values within a row with different superscripts differ significantly (*p* < 0.05). The values given in the table are the means calculated from 6 samples in each group.

**Table 3:** Parameters of mitochondrial function, measured using fluorespirometry method in the liver of newborn piglets (first day of life). Data are expressed as a % of the control group.

Item	Control	Fish Oil	Algal Oil	SE Pooled	*p*-Value
Basal respiration	100 ^a^	118 ^b^	111 ^b^	8.85	0.0351
ATP-linked respiration	100 ^a^	115 ^b^	110 ^b^	7.95	0.0259
Proton leak	100	108	100	8.24	0.2152
Maximal respiration	100 ^a^	120 ^b^	112 ^b^	9.53	0.0301
Spare respiratory capacity	100 ^a^	136 ^b^	111 ^ab^	10.25	0.0345

SE pooled—standard error of the mean for total animals; ^a,b^—values within a row with different superscripts differ significantly (*p* < 0.05). The values given in the table are the means calculated from 6 samples in each group.

**Table 4:** Pearson’s correlation coefficients (r) between parameters of mitochondrial function and DNA damage markers (expressed as cleaved abasic sites in DNA chain) in the liver of newborn piglets (first day of life).

Item	Marker of DNA Damage					
	8-oxo-G		εA		εC	
	r	*p*	r	*p*	r	*p*
Basal respiration	0.52	0.032	0.30	0.141	0.28	0.183
Maximal respiration	0.58	0.021	0.35	0.111	0.32	0.125
Proton leak	0.20	0.294	0.47	0.039	0.43	0.048
Spare respiratory capacity	−0.41	0.047	−0.15	0.415	−0.18	0.385

8-oxo-G—8-Oxoguanine; εA—ethenoadenine; εC—ethenocytosine. Pearson’s correlation coefficients were calculated based on total number of samples (*n* = 18).

**Table 5:** Nutritional composition of the standard diet for gilts, adjusted for early and late gestation stages.

Item	Day of Pregnancy	
	Till 90	90–114
Nutritional value (per kg diet)		
Crude protein, g	145	175
Lysine, g	8.0	10.0
Methionine	3.0	3.0
Methionine + Cystine	6.0	7.0
Threonine, g	6.0	6.5
Tryptophan, g	2.0	2.0
Crude fiber, g	50.0	36.0
Calcium, g	8.0	9.0
Total phosphorus, g	6.0	6.5
Digestible phosphorus, g	2.0	3.0
Vitamin A, IU	13,000	12,500
Vitamin E, mg	100	100
Vitamin D, IU	2000	2000
Metabolizable energy, MJ	12.0	13.0

**Table 6:** Fatty acid profile of the fish and algal oils used in the study.

Item	Norsan Omega-3 Arktis (per 10 mL)	Norsan Omega-3 Vegan (per 10 mL)
Components		
Fish oil	8.8 g	-
Algal oil	-	7.0 g
Olive oil	-	2.2 g
Fatty acid composition		
SFA	1.8 g	-
MUFA	3.9 g	-
PUFA	2.3 g	4.4 g
Omega-3	2.0 g	4.0 g
EPA	750 mg	1218 mg
DPA (docosapentaenoic acid)	95 mg	314 mg
DHA	975 mg	2316 mg

## Data Availability

The original contributions presented in this study are included in the article. Further inquiries can be directed to the corresponding authors.

## Abbreviations

AMPK	AMP-activated protein kinase
ATP	Adenosine triphosphate
BER	Base excision repair
DHA	Docosahexaenoic acid
DNA	Deoxyribonucleic acid
DPA	Docosapentaenoic acid
εA	1,N6-ethenoadenine
εC	3,N4-ethenocytosine
ETS	Electron transport system capacity
LC-PUFA n-3	Long-chain omega-3 polyunsaturated fatty acids
LEAK	Non-phosphorylating (proton leak) respiration
mtDNA	Mitochondrial DNA
OXPHOS	Oxidative phosphorylation capacity
PGC-1α	Peroxisome proliferator-activated receptor gamma coactivator 1-alpha
PPARα	Peroxisome proliferator-activated receptor alpha
RCR	Respiratory control ratio
ROS	Reactive oxygen species
SRC	Spare respiratory capacity
